# Impact of Land Use Change on the Habitat Quality Evolution in Three Gorges Reservoir Area, China

**DOI:** 10.3390/ijerph20043138

**Published:** 2023-02-10

**Authors:** Chunhua Peng, Yanhui Wang, Junwu Dong, Chong Huang

**Affiliations:** 1 Key Laboratory of 3-Dimensional Information Acquisition and Application, Ministry of Education, Capital Normal University, Beijing 100048, China; 2 State Key Laboratory of Resources and Environmental Information System, Institute of Geographic Sciences and Natural Resources Research, Chinese Academy of Sciences, Beijing 100101, China

**Keywords:** land use, habitat quality, InVEST model, multi-scale geographic weighted regression

## Abstract

Habitat quality (HQ) is an important indicator to characterize the level of biodiversity and ecosystem services, and can reflect the quality of the human living environment. Changes in land use can disturb regional HQ. Current research mostly focuses on assessing the good or bad quality of regional habitats, and less on the spatial response relationship between land use change and HQ, and even fewer studies on finely distinguishing the impact of land use types on HQ. Therefore, taking Three Gorges Reservoir Area (TGRA) of China as the study area, this paper first analyzes the land use change of study area by using the land use transfer matrix, land use rate model and landscape pattern index, and then combines the InVEST model with the multi-scale geographically weighted regression (MGWR) model to build a refined assessment framework to quantitatively assess the spatial and temporal evolution patterns of HQ, and then analyse in detail the spatial response relationship of each land use type change on the impact of HQ. The results showed that from 2000 to 2020, the land use in the TGRA shows a changing state of “urban expansion, cultivated land shrinkage, forest land growth, and grassland degradation”. With the change in land use, the habitat quality index (HQI) in the study area showed an “ increase first and then decline” change characteristics, and the HQ degradation was more obvious in the areas with intense human activities. The impact of land use change over the past 20 years on HQ in the TGRA has significant spatial and temporal heterogeneity, with changes in paddy and dryland having mainly negative impacts on HQ, and changes in sparse land, shrubland, and medium-cover grassland having mainly positive impacts on HQ. This paper mainly provides a research framework for refined assessment, and the results can provide scientific support for land planning and ecological protection in the TGRA, and the research methods and ideas can provide references for similar research.

## 1. Introduction

HQ refers to the ability of an ecosystem to provide a suitable living environment for individuals or populations [1]. It is a key indicator of the quality of the ecological environment, reflecting the level of regional biodiversity and ecosystem services, which is crucial to ensuring the ecological security of the region [2]. Land use change is an important factor causing changes in surface patterns and ecological environment. Blind expansion of land development and construction will lead to habitat degradation and fragmentation, thus affecting the material cycle and energy flow among habitat patches and bringing certain threats to the survival environment of species [3,4,5]. In recent years, with rapid urbanization, human activities have accelerated the change in land use patterns, which has led to changes in HQ [6]. Therefore, it is necessary to study the influence relationship between land use changes and HQ changes [7,8], which is important for achieving scientific land use planning and ecological environment protection.

In the early stages, HQ studies were mainly conducted on the species’ habitat quality in specific areas or the effects and impacts of different habitat conditions on a single species [9,10]. As research progressed, it became clear that the measurement and assessment of HQ required the integration of many characteristics of ecosystems [11]. Currently, most studies on habitat quality use the indicator system evaluation method [12] and the model method [13], but the indicator system method has not yet formed a unified evaluation index and standard, whereas the model method is relatively more accurate and data are easier to obtain, as well as less time consuming, less costly and more visible [14]. The habitat quality models mainly used at this stage are the Habitat Suitability Model (HSI) [15], the Maximum Entropy Model (Mx Ent) [16], and the InVEST model [17,18]. Among them, the InVEST model is mainly used for the assessment, management, and decision-making of ecosystem services, and the HQ module of this model is mostly used for habitat quality measurement in research [19,20]. The advantage of this model is that the sensitivity of habitats to threat sources is taken into account when assessing habitat suitability [21], and it is not limited by study time, scale, and area. It is currently the most frequently applied model and is widely used for HQ assessment, spatial and temporal variability, and future prediction in cities [22], watersheds [23], nature reserves [24], and coastal zones [25]. In addition, in studies related to the effects of land use change on HQ, the models such as contribution index [26], bivariate spatial autocorrelation [27], ordinary least square (OLS) [28], geodetector [29], geographically weighted regression (GWR) [30], multi-scale geographically weighted regression (MGWR) [31] were mainly used to quantify and assess the relationship between land use change and habitat quality change. Among them, the multi-scale geographically weighted regression model (MGWR) can not only analyze the spatial response relationships at different scales [32] but also better reveal the spatial response mode of land use change on habitat quality, providing an important means to explore the quantitative relationship between land use change and HQ in space.

The TGRA is located in the western part of China. It is a special ecological function area in the Yangtze River Basin and has an extremely important ecological status in China [33]. Since the completion of the Three Gorges Project, urban construction and economic development in the TGRA have surged, and the land use pattern of the region has changed dramatically, resulting in regional habitat fragmentation, habitat degradation and reduced biodiversity [34]. In recent years, with the implementation of a series of ecological projects, the ecological environment in some areas of the TGRA has improved significantly, but persistent human activity has made the human-land conflict still prominent, seriously threatening the HQ of the region [35]. Therefore, it is necessary to study the evolution characteristics of HQ under land use change. At present, there are numerous studies on land use and habitat quality in the TGRA [36,37], but few studies have shown the spatial relationship between land use types and habitat quality. In addition, among the available studies on HQ in the TGRA, most of them select local areas for study and cannot clarify the HQ status of the whole area [38,39,40]. At the same time, most of the studies have large differences in time points and lack evaluations for more recent periods [38,39]. Moreover, in the selection of parameters for HQ evaluation of the InVEST model, land use types are usually selected as habitat factors and threat factors, and the finer the classification of land use types and the smaller the scale of the raster data, the more accurate the evaluation results will be. However, most of the studies on HQ assessment in the TGRA focus on primary land use types [40], and the accuracy of the assessment results needs to be improved.

Therefore, this study takes the TGRA in China as the research object, based on the land use data from 2000 to 2020 (5 years’ timestep), First, we quantitatively analyzed the land use change in the TGRA by using a land use transfer matrix, a land use rate model, and a landscape pattern index. Secondly, we used the HQ module of InVEST software to assess the spatial and temporal change state of HQ in the TGRA, and analyzed the spatial differentiation characteristics of HQ with the help of the spatial autocorrelation model. Based on this, we finally used the MGWR model to quantitatively analyze the spatial response relationship between HQ change and land use change. The objectives of this study are to improve the accuracy of the TGRA habitat quality assessment results based on secondary class land use data, to clarify the characteristics of land use and HQ changes in the study area from 2000 to 2020, and to understand how land use changes affect the HQ of the region. This aims to provide a scientific basis for land use planning and ecological environmental protection in the TGRA, and may also provide reference values for similar studies in other areas.

## 2. Material and Methods

### 2.1. Study Area

**Figure 1:**
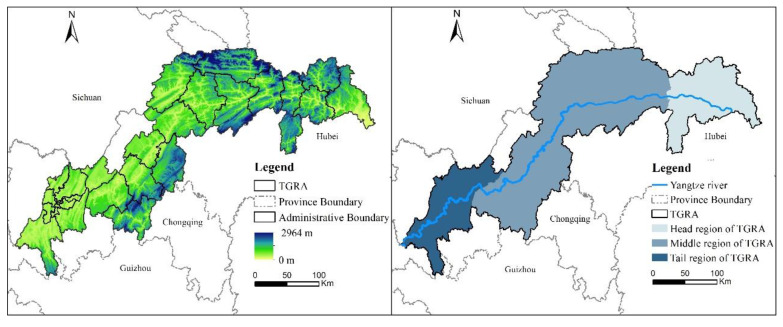
Location map of the TGRA.

The TGRA is located in southwest China and belongs to the lower part of the upper Yangtze River basin. The geographical coordinates are 28°32′ to 31°50′ north latitude and 106°20′ to 110°30′ east longitude, with an area of about 57,368 km^2^. The TGRA spans two provinces and cities, Chongqing and Hubei, and includes 26 districts and counties, 22 in Chongqing and 4 in Hubei (Figure 1). The TGRA is dominated by mountains and hills and is characterized by complex landforms and undulating terrain. The topography is characterized by a spatial distribution of “low in the southwest and high in the northeast”. In recent years, the expansion of human activities and the frequency of natural disasters have resulted in soil erosion, wetland degradation, and reduced biodiversity in the TGRA [33,37], resulting in significant changes to the surface cover and threatening the quality of the ecological environment.

As the TGRA has unique geographical characteristics that span two provinces and municipalities, we utilize the zoning standards of the “General Plan for the Near-Term and Medium-Term Agricultural and Rural Economic Development of the TGRA” to better reflect the spatial distribution pattern of habitat quality in this area. We divided the TGRA into three sections: the head region, the middle region, and the tail region (Figure 1).

### 2.2. Data Sources

In this study, five periods of land use data for 2000, 2005, 2010, 2015, and 2020 with a spatial resolution of 30m, were selected, all from the Data Center for Resources and Environmental Sciences, Chinese Academy of Sciences (RESDC) (https://www.resdc.cn). The remote sensing interpretation of the land use data for 2000, 2005, and 2010 were based on Landsat-TM/ETM remote sensing image data, and the land use data for 2015 and 2020 were based on Landsat 8 remote sensing image data. The land use classification mainly refers to the CNLUCC data classification system, including 6 primary classes and 17 secondary classes, with the accuracy of primary classification reaching over 93% and the accuracy of secondary classification reaching over 90% to meet the research needs [41].

This paper uses cultivated land, forest land, grassland, water area, construction land, and unused land as the mainland types for the study of land use change. Paddy land, dry land, forest land, shrub forest land, sparse forest land, other forest lands, high coverage grassland, medium coverage grassland, low coverage grassland, canal, lake, reservoir pond, tidal flat, urban land, rural residential area, other construction lands, and bare ground are used as the mainland types for calculating habitat quality. In addition, the natural breakpoint method was used for the threshold delineation of the relevant data values in this study.

### 2.3. Study Methods

The intensification of human activities is one of the important reasons for HQ degradation, and land use change can reflect the intensity of human activities. Currently, land use types are mostly used as model parameters to quantitatively evaluate HQ, but it is not deep enough to reveal the internal relationship between land use and HQ changes. Based on secondary land use data, this paper combines the InVEST model with the MGWR model to build a refined assessment framework, firstly, using the land use transfer matrix, land use rate model and landscape pattern index methods to quantitatively analyze the land use change, then using the HQ module of the InVEST model to assess the HQ of the study area, and then carry out spatial autocorrelation analysis and hotspot analysis on this basis. Finally, the MGWR model was used to analyze in detail the effects of land use change on HQ in the study area, and the spatial response of land use change on HQ evolution was discussed.

#### 2.3.1. Analysis of Land Use Change Methods

(1) Land Use Transfer Matrix

To reflect the structural characteristics of land use changes in the study area over time and the transformation relationship between the types. Based on the ArcGIS platform, this paper constructs land use transfer matrices for 2000–2005, 2005–2010, 2010–2015, 2015–2020, and 2000–2020 to compare and analyze the spatial and temporal changes of each land use type in the TGRA, and the calculated expressions are shown in (1):

(1) $$S_{ij}=\left[\begin{array}{cccc} S_{11} & S_{12} & \ldots & S_{1n}\\ S_{21} & S_{22} & \ldots & S_{2n}\\ \ldots & \ldots & \ldots & \ldots \\ S_{n1} & S_{n2} & \ldots & S_{nn} \end{array}\right]$$

In the formula: $S_{ij}$ is the land use status in the initial period and the end period, and *n* is the number of land use types.

(2) The Land Use Rate Model

The dynamic degree of land use can reflect the degree of change of a certain type of land use during the study period [40], expressed by the following formula:

(2) $$K_{i}=\frac{U_{b}-U_{a}}{U_{a}}\times \frac{1}{T}\times 100\%$$

In the formula: $K_{i}$ is the change rate of land use type *i* and the value larger, the human activities stronger. $U_{a}$ and $U_{b}$ are the areas of land use type *I* at the beginning and the end of the research, and *T* is the study duration.

(3) Landscape Pattern Index Analysis

The landscape pattern index can reflect the spatial configuration and structural composition within the landscape and is an important index to describe the landscape pattern [42]. In this study, the following landscape pattern indices were selected from the class level and landscape level to reflect the changing characteristics of land use in the TGRA. They include Patch Density (PD), Largest Patch Index (LPI), Landscape Shape Index (LSI), Mean Patch Area (AREA_MN), Contagion Index (CONTAG), Aggregation Index (AI) and Shannon’s Diversity Index (SHDI). The ecological significance and calculation of these landscape indicators are shown in Fragstats 4.2 software.

#### 2.3.2. InVEST Model

This study used the InVEST model to calculate HQ in the TGRA from 2000 to 2020. The principle of this calculation is to assume that habitat quality values are continuous variables belonging in the range from 0 to 1. The closer the value is to 1, the better the habitat quality, indicating better adaptation to species survival and better biodiversity. The closer the value is to 0, the worse the habitat quality, indicating that the ecosystem is less able to support the survival and reproduction of species, which is detrimental to the maintenance of biodiversity in the region [43]. In this paper, HQ is calculated based on the sensitivity of different land use types and the intensity, location, and maximum impact distance of different threat sources using land use data and drawing on relevant studies [23,37]. The calculation equation is as follows:

(3) $$Q_{xj}=H_{j}\left[1-\left(\frac{D_{xj^{z}}}{D_{xj^{z}}+K^{z}}\right)\right]$$

In the formula: $Q_{xj}$ represents the habitat quality value corresponding to grid pixel x in land use type j; $H_{j}$ represents the habitat adaptability value corresponding to land use type j; z is a constant, and its value is usually 2.5; $D_{xj}$ represents the weighted average of various threat sources corresponding to grid pixel x in land use type j; k represents half-saturated constant, usually the default value is 0.5, the threat source. The parameters and sensitivity are shown in Table 1 and Table 2.

#### 2.3.3. Spatial Auto-Correlation and Hot Spot Analysis

Spatial auto-correlation is a quantitative description of the similarity of attribute values or spatial association patterns of adjacent units in space, and it is used to determine the degree of spatial association of an attribute between neighbouring units. There are two types of spatial autocorrelation: global spatial auto-correlation and local spatial auto-correlation. The global spatial auto-correlation Moran’s I was used in this study to describe the spatial correlation of habitat quality in the study area. Moran’s I values ranged from [−1, 1], with values greater than 0 indicating positive correlation and less than 0 indicating negative correlation, and its absolute value indicating the strength of correlation. The hotspot analysis method was used in this study to investigate the local spatial clustering characteristics of HQ and to determine whether there was high-value or low-value aggregation. The specific formulae for the two spatial analysis methods are mainly referred to in the literature [44].

#### 2.3.4. Multiscale Geographically Weighted Regression (MGWR) Model

Multiscale geographically weighted regression (MGWR) models allow for different levels of spatial smoothing for each variable and use different bandwidths for each independent variable to determine the spatial scale at which each dependent variable acts [32]. According to related research, the MGWR model is more consistent with geographic process spatial heterogeneity than the geographically weighted regression (GWR) and multiple linear regression (OSL) models [28]. The amount of change in habitat quality was chosen as the dependent variable for this study, and the amount of change in land use type area was chosen as the independent variable.

(4) $$y_{i}=\sum_{i=1}^{k}\beta_{bwj}\left(u_{i},v_{i}\right)x_{ij}+\varepsilon_{i}$$

In the formula: $y_{i}$ is the dependent variable; $x_{ij}$ is the  j independent variable, representing the local parameter estimation of the kth independent variable of sample i; bwj is the bandwidth used by the regression coefficient of the jth variable, the relationship between the independent variable and the dependent variable allows change space; $\left(u_{i},v_{i}\right)$ are the coordinates of the geographical location of sample i; $\beta_{bwj}\left(u_{i},v_{i}\right)$ is the regression coefficient of the independent variable; $\varepsilon_{i}$ is the random error.

## 3. Results

### 3.1. Temporal and Spatial Variation Characteristics of Land Use in the TGRA

#### 3.1.1. Land Use Change Analysis

**Figure 2:**
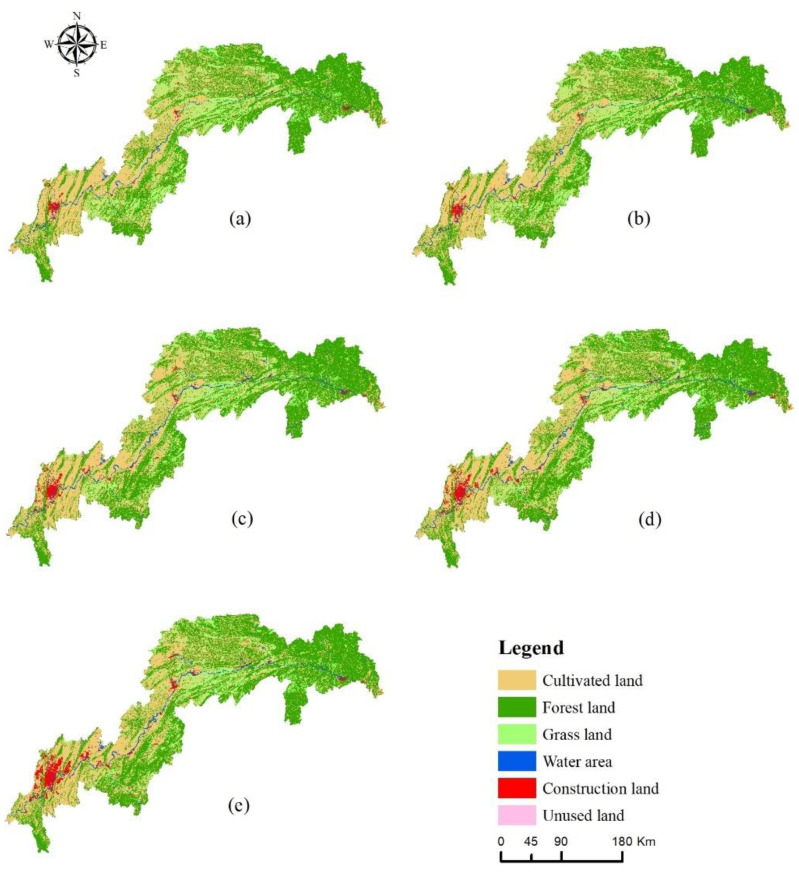
Spatial distribution of land use types from 2000 to 2020 ((**a**) 2000, (**b**) 2005, (**c**) 2010, (**d**) 2015, (**e**) 2020).

In the past 20 years, the spatial distribution of land use in the TGRA has been distinct (Figure 2). Forest land and cultivated land are the main land use types in the study area, with the combined area of the two exceeding 84% of the total study area in all periods. Forest land is mainly distributed in the northeastern and southeastern mountain ranges of the TGRA, whereas cultivated land is mainly distributed in the northeastern and western regions of the TGRA. Grassland is relatively scattered, mainly in the southwestern and north-central regions of the study area. Construction land is concentrated in the western region and along the river and shows a clear trend of expansion over time. The water area runs from east to west along the Yangtze River throughout the study area. Unused land is spatially scattered and very small in size, and from 2000 to 2020, the area of land use types in the TGRA changed significantly (Table 3). The area of cultivated land, grassland, and unused land all decreased, and the land use dynamic attitude was all negative, with the area of grassland decreasing the most, by 1677.72 km^2^. The area of forestland, water, and construction land all increased, and the overall land use dynamic was positive, with the largest increase in the area of construction land, which increased by 1769.18 km^2^.

#### 3.1.2. Land Use Transfer Analysis

Table 4 shows the transfer between land use types in the TGRA in different periods. From 2000 to 2005, the area of land use transfer was 1303.15 km^2^, accounting for 2.27% of the total area of the TGRA. Among them, the area of cultivated land, grassland, and unused land all decreased, with cultivated land decreasing the most, by 654.69 km^2^, mainly turning into forest land, grassland, and construction land. The area of forest land increased the most in this period by 561.76 km^2^. From 2005 to 2010, the conversion between land use types in the study area increased significantly, with a total conversion of 2899.16 km^2^. Grassland decreased the most, by 1273.66 km^2^ in this period, mainly transformed into forest land and cultivated land. At the same time, the area of forest land increased the most, by 1128.23 km^2^, mainly from grassland and cultivated land. The area of land for construction also increased by 429.55 km^2^, mainly from cultivated land. From 2010 to 2015, the area of land use transfer accounted for only 1.41% of the total area of the study area, a substantial reduction in the degree of conversion compared to the previous two periods. During this period, the largest area of cultivated land outflow was 457.95 km^2^, mainly converted to forest land and construction land. This was followed by a transfer out of forest land of 236.15 km^2^, mainly to cultivated land and construction land, and the largest area of construction land was transferred in during this period at 251.49 km^2^; From 2015 to 2020, the land use conversion in the study area was drastic, with a conversion area of 4581.64 km^2^. The land use conversion in this period is similar to that in the previous period. Cultivated land, forest land, grassland, and unused land continued to flow out, with the largest area of cultivated land still being converted out at 2083.62 km^2^, mainly to forest land and construction land. At the same time, the area of land for construction increased the most in the period by 676.62 km^2^, mainly from the transfer of cultivated land.

Looking at the entire period from 2000 to 2020, the land use transfer area in the TGRA totals 6723.90 km^2^, accounting for 11.73% of the total area of the study area. In terms of the conversion of each land type, the area of forest land, water area, and construction land increased, although the area of cultivated land, grassland, and unused land decreased. Forest land was mainly converted from cultivated land and grassland, which is closely related to the policy of returning cultivated land to forest and grass, and construction land was mainly converted from cultivated land, especially the expansion trend of construction land centered on the western urban area of the study area is more significant. In terms of the relative amount of transfer out and in, the overall area of grassland decreased and was the largest, at 1500.66 km^2^, followed by an overall decrease of 913.43 km^2^ in cultivated land, whereas the overall area of construction land increased and was the largest, followed by forest land, with an area of 1313.82 km^2^ and 764.63 km^2^ respectively.

#### 3.1.3. Landscape Pattern Index Change Analysis

**Figure 3:**
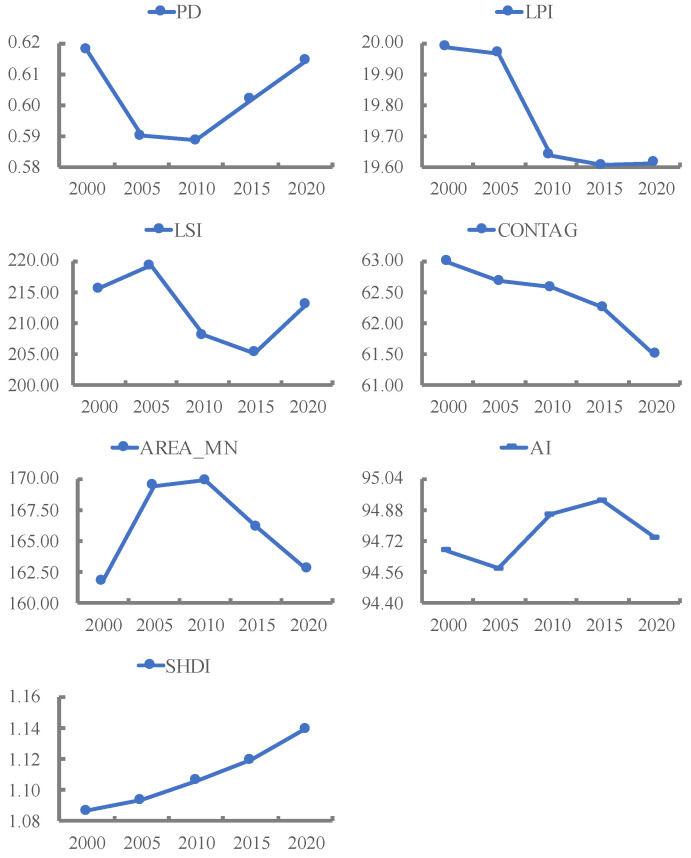
Index change of landscape pattern at the landscape level.

The change in land use landscape pattern index at the landscape level in the TGRA in the past 20 years is shown in Figure 3. Taking 2010 as the turning point, the PD of the study area first declines and then rises, and AREA_MN first rises and then falls, indicating that the spatial heterogeneity and fragmentation of the study area’s landscape intensified after 2010. From 2000 to 2020, LPI and CONTAG declined significantly, decreasing by 0.37 and 1.49, respectively, indicating that the pattern of landscape change has a greater influence on the largest patches, and the increase in landscape fragmentation also leads to the decrease in landscape spreading. LSI showed a change characteristic of rising, then falling, then rising again, reflecting the gradual tendency of landscape morphology to be irregular and diversified. During the study period, the AI showed a trend of decreasing, then increasing, and then decreasing, with a sharp decrease in AI after 2015, indicating that the landscape patches in the study area gradually separated and the ability of patches to aggregate weakened. SHDI showed a continuously increasing trend from 1.09 to 1.14, which is at a high level overall, reflecting the high landscape richness of the TGRA and the strong balance of each landscape.

**Figure 4:**
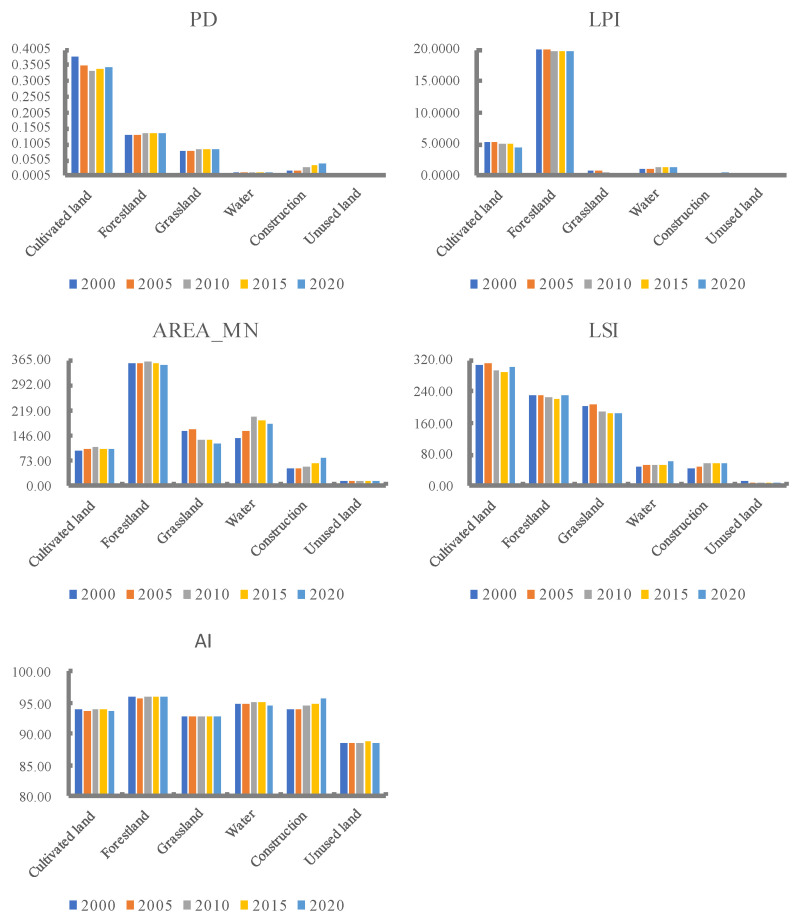
Index change of landscape pattern at class level.

The changes in the landscape pattern of land use types can be well explained at the landscape class level. The changes in land use types at the landscape class level in the TGRA in the last 20 years are shown in Figure 4. From 2000 to 2020, the area of cultivated land decreased, the PD, LPI, LSI, and AI of cultivated land decreased, and the AREA_MN increased, showing that the cultivated land patches were divided, the number of dominant cultivated land patches decreased, and the agglomeration decreased. The LPI, AREA_MN, and AI of forestland were the largest in the last 20 years, indicating that forestland is the dominant landscape in the reservoir area with high spatial aggregation among patches. Grassland shows a decreasing trend in all landscape pattern indices except PD, where LPI, AREA_MN, and LSI are significantly decreasing, indicating that grassland fragmentation is increasing, landscape dominance is decreasing, aggregation is weakening, and larger patches are divided. The overall development of the water area is good, and the patch area has increased, AREA_MN, LSI, and LPI have increased by 39.46, 10.2, and 0.49, respectively. With the rapid expansion of construction land, each landscape pattern index of construction land has continued to rise, among which AREA_MN and LSI have increased by 30.37 and 15.49 respectively in the past 20 years, indicating that with the accelerated development of urbanization construction, the built-up area continues to grow, becoming more and more aggregated and highly connected, and the spatial pattern changes. The area of unused land continues to decrease, and its PD, AI, LSP, and other landscape indices all show a certain degree of decline, and the landscape patches become more dispersed and the degree of aggregation gradually decreases.

### 3.2. Temporal and Spatial Variation Characteristics of Habitat Quality in the TGRA

#### 3.2.1. Temporal Variation of HQ

**Figure 5:**
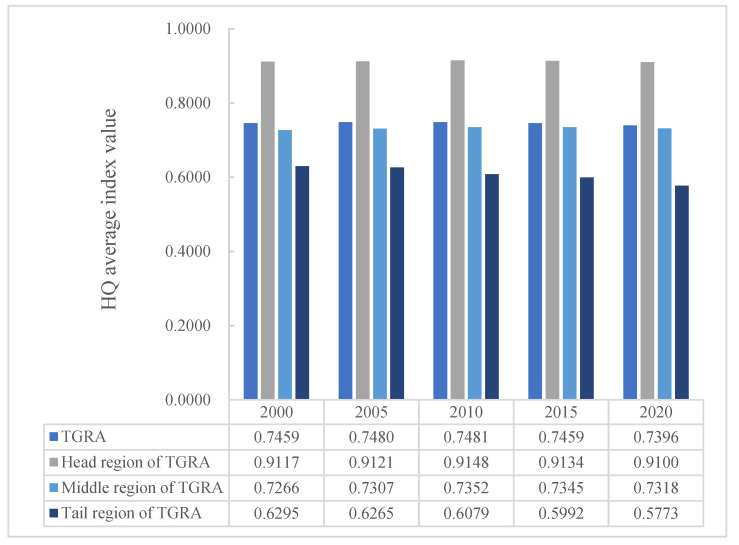
The average value of habitat quality in the TGRA from 2000 to 2020.

The Habitat Quality Index (HQI) is a value that varies continuously from 0 to 1. The closer the value is to 1, the better the habitat quality is, and vice versa. In this study, the habitat quality results based on the InVEST model were categorized into five grades, and the mean value of HQI for each year and the area ratio of each grade of HQ were calculated (Figure 5, Table 5). Figure 5 shows the average HQ values of TGRA at each time point from 2000 to 2020. The HQ values were 0.7459, 0.7480, 0.7481, 0.7459, and 0.7396 in that order, showing an overall trend of increasing and then decreasing. By 2020, the HQ value has decreased by 0.0063. Overall, the habitat quality of the TGRA is relatively high, but there is a trend toward degradation. Based on the statistical results for each class, the HQ of the TGRA is generally dominated by the high class, with the sum of the proportion of the area of medium-high class and high class exceeding 50% in each period. By 2020, the habitat quality area of medium-high class increased by 466.06 km^2^, the area of medium-low grade has decreased by 492.95 km^2^, the area of habitat quality varied significantly between the two classes. Over the last 20 years, the area of medium habitat quality has decreased by 3.65% and the area of low habitat quality has increased by 2.3%.

#### 3.2.2. Spatial Variation of HQ

**Figure 6:**
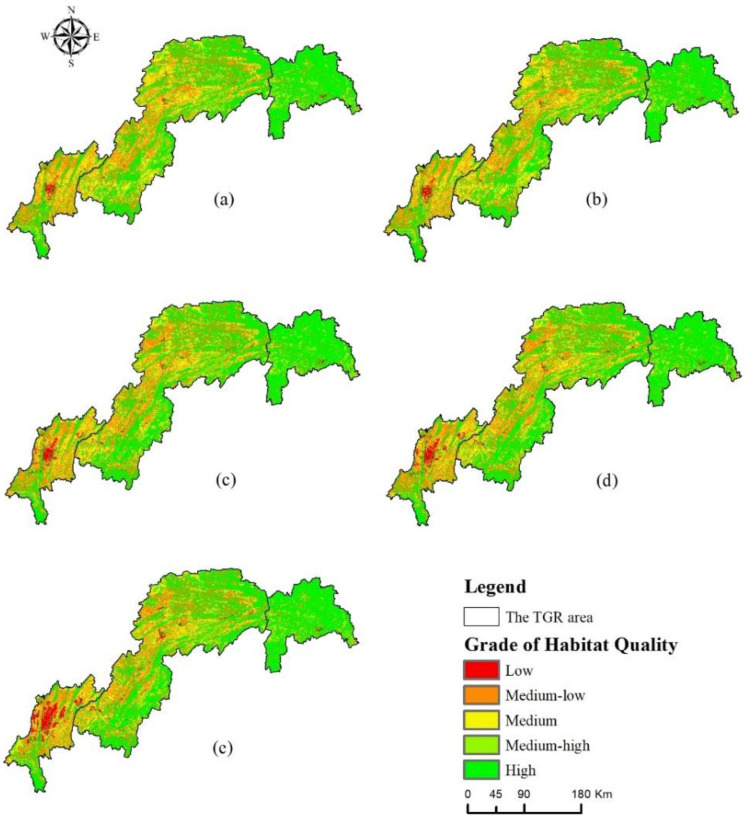
Spatial distribution of HQ level from 2000 to 2020 ((**a**) 2000, (**b**) 2005, (**c**) 2010, (**d**) 2015, (**e**) 2020).

According to the divisional statistical results (Figure 5), the head region of TGRA had the highest mean value of HQI from 2000 to 2020, with values around 0.91 in all years, which was greater than the overall habitat quality level of the study area. The mean value of HQI in the middle region of TGRA generally remained around 0.74, which was consistent with the overall habitat quality of the study area. The mean value of HQI in the tail region of TGRA meant the value was lower than the overall level of the study area, had a decreasing trend year by year, and would drop to about 0.58 by 2020. The spatial distribution of each habitat quality class is depicted in Figure 6. The habitat quality in the study area changed significantly from 2000 to 2020, and generally showed a spatial distribution characteristic of “high in the northeast and low in the southwest”. The low level of habitat quality was mainly concentrated in the central urban area in the tail area of TGRA and gradually spread from the urban area to the surrounding areas. The land use in this area was mainly construction land, and the overall habitat quality was poor. The middle high and high-grade habitat quality was concentrated in the head area of TGRA, as well as the mountainous areas in the northeast and southeast of the middle region of TGRA, which were mainly forest lands and rich in biodiversity. The medium-grade habitat quality was spatially distributed in a contiguous pattern, mostly concentrated in the eastern and western parts of the middle area of the TGRA and the northeastern part of the tail area of the TGRA.

**Figure 7:**
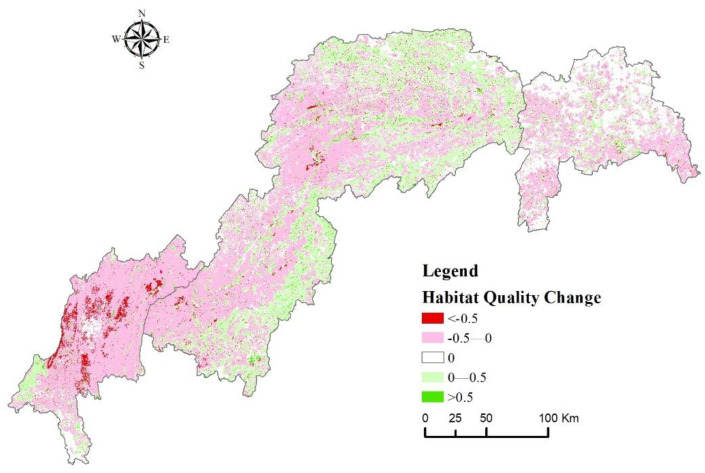
Spatial distribution of habitat quality change difference from 2000 to 2020.

To more intuitively reflect the spatial and temporal characteristics of HQ in the TGRA, this study calculated the difference between the habitat quality indices in 2000 and 2020 and divided the resulting values into five categories. The statistical results and spatial distribution are shown in Table 6 and Figure 7. The area of slightly degradation habitat quality was the largest, accounting for 47.19% of the total area of the TGRA, and accounting for 7.49%, 27.15% and 12.55% of the area of the head, middle and tail of the TGRA respectively. Habitat quality stabilization areas accounted for 25.11% of the area, mainly in the middle parts of the TGRA. Slightly improved and significantly improved habitat quality areas were mainly located in the Yangtze River watershed and mountainous areas in the middle region, accounting for 18.56% and 1.57% of the total area respectively. In addition, the total area of significant degradation habitat quality in the study area was only 3%, mainly in the areas with strong human activities in the middle and tail of the TGRA, accounting for 1.39% and 1.35% respectively.

#### 3.2.3. Spatial Auto-Correlation Analysis of HQ Value

**Figure 8:**
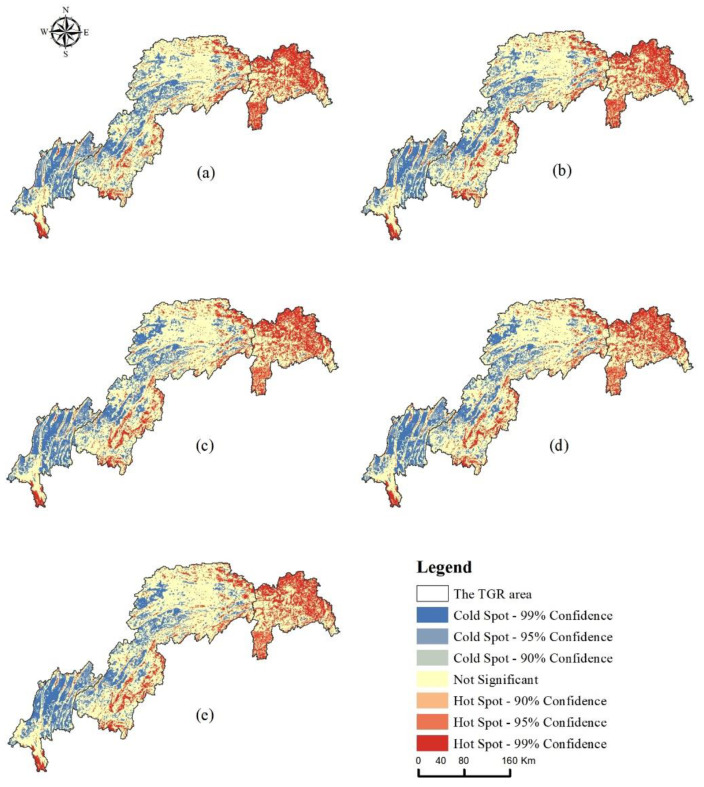
Spatial Distribution of Cold and Hot Spots of Habitat Quality in the TGRA ((**a**) 2000, (**b**) 2005, (**c**) 2010, (**d**) 2015, (**e**) 2020).

This paper uses spatial auto-correlation and cold and hot spots to analyze the spatial heterogeneity of HQ in the study area. First, the global auto-correlation Moran’s I was used to exploring the spatial correlation of HQ in the study area. The Moran’s I indices for 2000, 2005, 2010, 2015, and 2020 were 0.762, 0.769, 0.778, 0.779, and 0.787, respectively, with continuously increasing index values and P values of 0, indicating a significant spatial clustering effect of HQ. The spatial distribution of HQ hot spots in the TGRA is shown in Figure 8, with similar spatial distribution characteristics in all periods, showing a spatial pattern of “high in the east and low in the west”. Hot spots were mainly distributed in the northeastern region of the TGRA’s head and the TGRA’s middle and were most concentrated in the head region, where forestland and grassland were the mainland types. Cold spots were mainly located in the northwestern part of the TGRA’s tail and the TGRA’s middle, and were mostly concentrated in the tail area, with cultivated land and construction land being the main land types in the area. In terms of temporal changes, from 2000 to 2020, the area of hotspots with 99% confidence in the study area decreased by 1.98%, whereas the area of hot spots with 95% and 90% confidence increased by 1.86% and 0.51% respectively. At the same time, the area of cold spots with 99%, 95%, and 90% confidence levels decreased by 0.21%, 0.97%, and 0.4% respectively. Therefore, the differences in the spatial distribution of hot and cold spots in the study area were closely related to the distribution pattern of land use types. High habitat quality values were mainly clustered in areas with dense vegetation distribution, although low habitat quality values were mainly concentrated in areas with cultivated land and construction land distribution.

### 3.3. Impact of Land Use Change on HQ

#### 3.3.1. Analysis of HQ Change Based on Land Use Change

**Figure 9:**
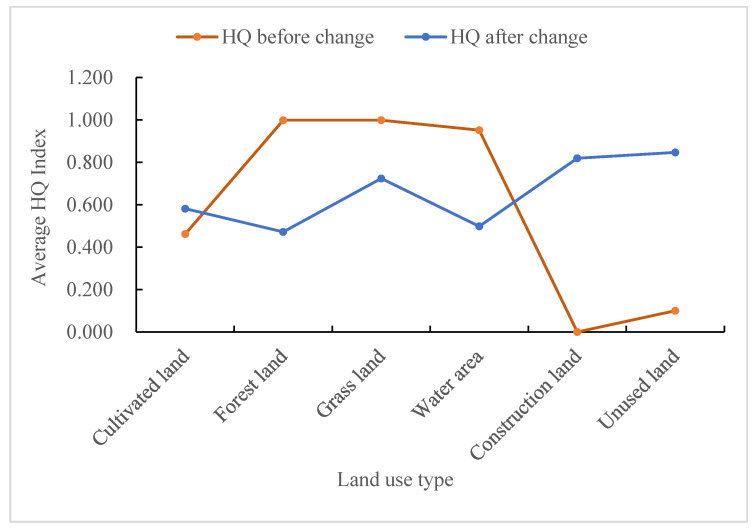
HQ index values before and after land use type change from 2000 to 2020.

To explore the impact of land use change on HQ, this study counted the HQI values of each land use type before and after the change from 2000 to 2020 (Figure 9). Statistically, the habitat quality in the areas where cultivated land, construction land, and unused land were located was low before land use conversion in the study area. The HQ improved significantly after the conversion, and the HQI value of construction land increased significantly from 0 to 0.819 after the conversion. HQ decreased in the areas where the conversion of forest land, grassland, and water occurred, with the greatest decrease in habitat quality in the converted area of forest land, from 0.998 to 0.472, although the decrease in habitat quality in the converted area of grassland was relatively small.

**Figure 10:**
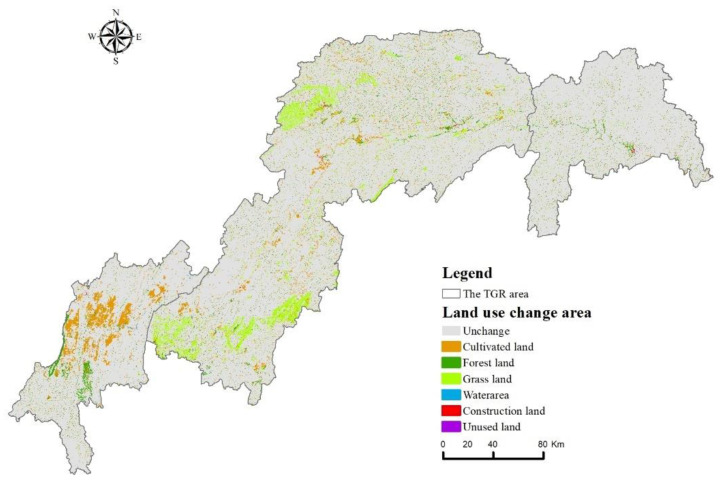
Land use change area from 2000 to 2020.

In terms of spatial distribution (Figure 10), the area of cultivated land, forest land, and grassland in the study area have undergone the largest change. Among them, the area where cultivated land had changed was mainly distributed in the middle and tail of the TGRA, with an area of 1672.77 km^2^ and 1132.97 km^2^ respectively. In the middle of the TGRA, the cultivated land was mainly converted to forest land and grassland, whereas in the tail area, the cultivated land was mainly converted to construction land. Forest land was mainly converted to cultivated land and was concentrated in the middle of the TGRA, with an area of 894.59 km^2^. Grassland conversion areas were mainly located in the central part of the TGRA, with a conversion area of 1985.86 km^2^, mainly converted into forest land and cultivated land. Water areas were converted more in the middle and tail areas of the TGRA and were mainly converted into cultivated land. Construction land was mainly converted to water in the head and middle regions of the TGRA, and mainly converted to cultivated land in the tail part of the TGRA, and the quality of the habitat was greatly improved after the conversion. Overall, there were significant differences in the effects of different land use orientations on habitat quality in the study area, and the degree of impact on HQ varied from region to region.

#### 3.3.2. Response Analysis of HQ to Land Use Change

Changes in land use have different degrees of impact on HQ, and the response of HQ to changes in land use is also temporally and spatially heterogeneous. To further identify in detail the spatial response pattern of HQ change to land use change in the study area, this study takes 2000–2020 as the research period, divides the study area into 1 km × 1 km research units, takes the habitat quality change as the dependent variable, and takes the area change of the secondary land use type as the independent variable, using the MGWR model to carry out regression analysis. According to statistics, the changing area of secondary land use types in the study area from 2000 to 2020 was 13,262.81 km^2^, of which the changing area of paddy field, dry land, forest land, sparse forest land, shrub forest, and medium coverage grassland accounted for 95.89% of the total change area. It can represent the overall characteristics of land use change in the study area. As a result, the above six types of secondary land types with large area changes were chosen as the primary research objects for this study. The results showed that the MGWR model fitted well, with an R^2^ adjusted value of goodness of fit of 0.60.

**Figure 11:**
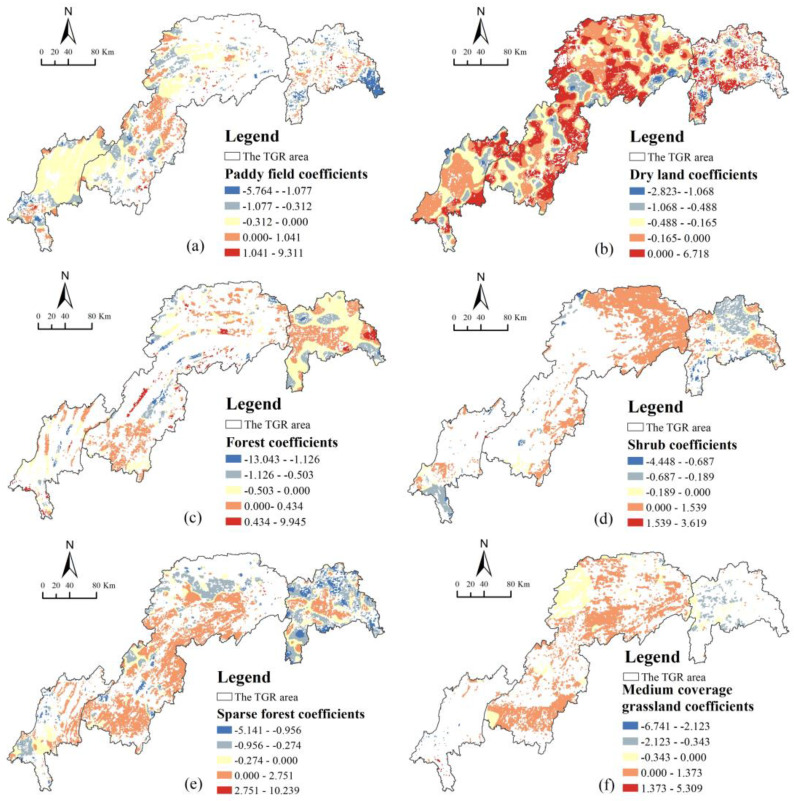
Spatial distribution of regression coefficients of MGWR model from 2000 to 2020 ((**a**) paddy field, (**b**) dry land, (**c**) forest land, (**d**) shrubland, (**e**) sparse forest land, (**f**) medium-coverage grassland. Also the white area is the background).

The spatial response of habitat quality change to different land use changes from 2000 to 2020 is shown in Figure 11. As shown in Figure 11a, HQ change was primarily negatively correlated with paddy field area change, with a negative regression coefficient accounting for 73.89% of the area. The area with the strongest negative correlation was located in the eastern part of the TGRA’s head region, where paddy fields had been primarily converted to forest land and sparse forest land, and HQ had been improved. Paddy fields have primarily been converted into sparse forest land and medium-cover grassland in the area with a strong negative correlation between the middle and tail regions of TGRA. Locally positively correlated areas were spatially dispersed, with paddy fields primarily converted to other construction lands in the TGRA’s head region, to dry land and other construction lands in the TGRA’s middle region, and to other construction lands, urban land, and dry land in the TGRA’s tail region, resulting in HQ degradation.

As shown in Figure 11b, there was a general negative correlation between HQ change and dry land area change, with dry land in the head region of the TGRA primarily transformed into paddy fields and various types of forest land, in the middle region of the TGRA primarily transformed into various types of forest land, paddy fields, medium-coverage grassland, and water areas, and in the tail region of the TGRA transformed into various types of forest land and paddy fields. The locally positively correlated areas were primarily located in the TGRA’s middle region and were primarily converted to rural residential areas and other construction lands, whereas they were primarily converted to urban land and other construction lands in the TGRA’s head and tail regions, resulting in a decrease in HQ.

There was a positive correlation between HQ change and forested land area change, as shown in Figure 11c, with the highest correlation in the eastern part of the TGRA’s head region, where forest land was primarily converted to river canals, sparse forest land, and dry land, resulting in HQ degradation. The distribution was primarily along the mountain range in a strip in the middle and tail regions of the TGRA, and the conversion was primarily to dryland, sparse forest land, and medium cover grassland in the middle region of the TGRA, and the conversion was primarily to dry land, paddy field, construction land, and sparse forest land in the tail region of the TGRA.

As illustrated in Figure 11d, there was a significant positive correlation between HQ change and shrubland area change, with 72.02% of the positive correlation located primarily in the eastern part of the TGRA’s middle region, where shrubland was primarily converted to dry land and medium-cover grassland, resulting in HQ degradation. A small number of positively correlated areas were primarily converted to dry land and sparse forest land in the TGRA’s head region, and dry land, paddy fields, and urban land in the TGRA’s tail region. Furthermore, in areas with a local negative correlation at the TGRA’s head and tail regions, shrubland was primarily converted to forest land, and the HQ improved.

According to Figure 11e, HQ changes were primarily positively correlated with changes in the area of sparse forest land, with the positively correlated areas primarily located in the south-western part of the TGRA’s middle region, where sparse forest land was primarily converted to paddy, dry land, grassland, and construction land, and HQ decreased. Some of the positively correlated areas were primarily converted to dry land in the TGRA’s head region, and to dry land, paddy land, and other built-up lands in the TGRA’s tail region. Furthermore, some of the negatively correlated areas were converted primarily to river canals and forest land at the TGRA’s head, and primarily to forest land and shrubland at the TGRA’s middle and tail, and the HQ was improved.

The change in HQ is significantly positively correlated with the change in the area of medium-coverage grassland, as shown in Figure 11f, with 69.58% of the positively correlated areas concentrated in the eastern and southern parts of the middle region of TGRA, where medium-coverage grassland was primarily converted to dry land, rural settlements, and other construction lands, resulting in a decrease in HQ. Some of the negatively correlated areas could be found in the northwestern and central parts of the TGRA. In the TGRA’s head region, high-cover grassland was primarily converted into sparse forest land, forest land, and other forest lands, whereas in the TGRA’s middle region, it was primarily converted into paddy fields, various types of forest land, and high-cover grassland, with a small portion, converted into rivers, canals, and reservoir ponds, which improved the HQ.

## 4. Discussion

### 4.1. Impact of Land Use Change on HQ

The study’s findings show that overall HQI values in the TGRA fluctuated less between periods (Figure 5), but in terms of zoning, HQ was highest in the TGRA’s head region, whereas HQ in the TGRA’s middle region remained consistent with that of the study area as a whole, with a slight decline in both areas after 2010. HQ in the TGRA tail region was not only low but also decreasing year on year, which was linked to urban expansion. With the completion of the Three Gorges Project, the development of urban construction, and the implementation of policies such as returning farmland to forests and grasses, the land use types and HQ in the study area have undergone significant changes in the last 20 years. In this paper, a study of the impact of primary land use type changes on HQ (Figure 9) discovered that decreasing the area of cultivated land, construction land, and unused land increased the level of HQ in the region whereas decreasing the area of forest land, grassland, and water area decreased HQ. Changes in HQ were significantly negatively correlated with changes in the area of paddy fields and dry land, and significantly positively correlated with changes in the area of sparse forest land, shrub land, and medium-cover grassland in the study of changes in secondary land use types and changes in HQ (Figure 11), which was similar to the pattern of HQ changes in primary land types.

Furthermore, the areas with significant HQ degradation in the study area were primarily located in areas with intensive human activities, such as the urban areas in the reservoir’s central part and some areas along the Yangtze River (Figure 7), and the land use types in these areas were primarily urban land, rural settlements, and other construction lands, which was consistent with the spatial distribution pattern of the current land use areas (Figure 2). The landscape pattern of the various land use types in the study area has changed significantly over the last 20 years, with cultivated land and grassland exhibiting spatial fragmentation, complex shapes, and increased fragmentation, and forest land and building land exhibiting increased patch size and spatial aggregation. Furthermore, changes in landscape patterns have affected the spatial pattern of HQ to some extent, and furthering landscape fragmentation will intensify the spatial differentiation of HQ, leading to a reduction in biodiversity.

### 4.2. Habitat Quality Improvement and Ecological Conservation Strategies

The quality of habitat is critical to ensuring regional ecological security, preserving biodiversity, and achieving sustainable regional development. The rapid socioeconomic development of areas along the Yangtze River, as well as the significant increase in construction land, has resulted in a significant decline in HQ in urban areas over the last 20 years. The study discovered that 2010 was a critical time point for HQ changes in the TGRA, and that HQ has declined significantly since then. The three regions of the segment showed similar characteristics of HQ changes, and studies have shown that human-land conflict caused by urban expansion was an important factor affecting HQ in the TGRA [40]. At the same time, this study discovered significant variability in HQ in different areas of the TGRA, with a stepwise change in the head, middle, and tail areas, with habitat quality in the tail area being lower than the overall level of the study area, HQ in the middle area was close to the overall level of the study area, and HQ in the head area was the best, far above the overall level of the study area. In addition, studies on the relationship between habitat quality change and land use change have shown that the dominant land types for habitat quality change in different regions were different.

As a result, ecological restoration and enhancement work in the study area’s areas with degraded HQ should be site-specific. The TGRA’s head region is primarily composed of paddy fields and forestlands, with paddy fields primarily converted to other construction land and forestlands converted to dry land and river canals, as well as a conversion of high vegetation cover forestlands to low vegetation cover forestlands. As a result, in the head region, it is necessary to control cultivated land cultivation, and strengthen the implementation of the policy of returning farmland to forest agroforestry. In the middle region, paddy land has primarily converted to dry land, and dry land has primarily converted to construction land; forested land and shrubland have primarily converted to dry land and open forest land; open forest land has primarily converted to cultivated land, grassland, and various types of construction land; and grassland has primarily converted to dry land, rural settlements, and other construction lands. The study discovered that the direction of change in land types in the middle area has been complex, with the overall change favouring dry land. As a result, it is critical to strengthen the project of returning farmland to forest and grassland, optimize the ecological functions of the land, strengthen forest and grassland protection, and strictly limit the disorderly expansion of rural settlements. Because forest land has primarily converted into cultivated land and construction land in the tail region, and cultivated land has primarily converted into construction land, the tail region should prioritize protecting natural landscapes such as forest land, reducing damage to them from human activities, strictly controlling the blind expansion of construction land, and strengthening control over the growth of town boundaries. To achieve an overall improvement in HQ in the TGRA, ecological protection and restoration work must be carried out on a site-specific basis, and the various management departments must co-ordinate the relationship between ecological, production, and living spaces in the TGRA, as well as promote stable, high-quality, and sustainable habitat quality improvement in the TGRA.

### 4.3. Research Contribution and Shortcomings

The contribution of this study is to provide an overall research framework for a refined assessment of the effects of land use change on HQ. We assessed HQ in the study area mainly based on secondary land use type data and explored the spatial response relationships of secondary land use type changes on HQ. Because different land use types have different directions of influence on HQ, and the classification of land use has a scale effect, the finer the classification of land use types, the more accurate the assessment results of HQ. Relevant studies have shown that using different classification levels of land use type data, the results of evaluating HQ based on the same method are significantly different, and the results of evaluating only the first-level land use type are somewhat one-sided [29,45]. Therefore, it is more valuable to explore the impact of land use change on HQ by secondary class of land use types. In addition, in the study of HQ in the TGRA, there is a lack of studies that integrate “long time, small scale, fine-grained, and holistic” [37,39,40,46] concepts. The contribution of this study is to integrate the above issues and update the study on the impact of land use change on HQ in the TGRA.

## 5. Conclusions

This study takes the TGRA in China as the study area and uses the InVEST model to measure the HQ level in the study area for the past 20 years by selecting five phases of land use data, combining the land use transfer matrix, land use rate model, and landscape pattern to analyze the spatial and temporal evolution characteristics of habitat quality from a directional and quantitative perspective, and exploring the response state of HQ changes to each land use change spatially based on the MGWR model. The results show that from 2000 to 2020, the land use in the TGRA shows a changing state of “urban expansion, cultivated land shrinkage, forest land growth, and grassland degradation”; The direction of land use transfer is mainly manifested in the transfer of cultivated land and grassland to forest land and construction land, which made the patch fragmentation of cultivated land and grassland deepen, whereas the patch aggregation degree of forest land and construction land increased and the connectivity became better; In the past 20 years, the HQ in the study area has shown a trend of “rising first and then declining”. The high values of HQI are mainly concentrated in the mountainous areas in the northeast of the TGRA, and low values mainly in the urban areas in the western part of the TGRA and along the Yangtze River. The impact of each land use type on habitat quality has significant spatial and temporal heterogeneity, with changes in the area of paddy fields and drylands negatively correlated with changes in HQ, and changes in the area of shrub lands, sparse forest lands and medium-cover grasslands significantly positively correlated with changes in HQ.

## Figures and Tables

**Table 1:** Weight assignment and maximum influence of threat factors.

Threat Factors	Maximum Impact Distance/km	Weight	Decay Type
Paddy field	1	0.5	Linear
Dryland	2	0.6	Linear
Urban land	10	1	Exponential
Rural residential area	6	0.7	Exponential
Other construction lands	8	1	Exponential
Unused land	2	0.6	Linear

**Table 2:** Sensitivity parameters of land use types to threat factors.

Land Use Type	Habitat Suitability	Sensitivity to Threat Sources					
		Paddy Field	Dryland	Urban Land	Rural Residential Area	Other Construction Land	Unused Land
Paddy field	0.6	0	0.3	0.6	0.6	0.4	0.3
Dryland	0.4	0.3	0	0.6	0.6	0.4	0.3
Forest	1	0.5	0.5	1	0.8	0.7	0.4
Shrub	1	0.5	0.6	0.9	0.6	0.6	0.3
Sparse forest	1	0.6	0.7	1	0.9	0.65	0.4
Other forests	1	0.9	0.9	1	0.8	0.70	0.4
High coverage grassland	0.8	0.4	0.4	0.7	0.6	0.4	0.7
Medium coverage grassland	0.7	0.5	0.5	0.7	0.6	0.4	0.7
Low coverage grassland	0.6	0.5	0.5	0.6	0.6	0.5	0.7
Canal	1	0.65	0.65	0.70	0.55	0.45	0.3
Lake	1	0.70	0.70	0.9	0.75	0.50	0.3
Reservoirs pond	1	0.70	0.70	0.9	0.75	0.50	0.3
Tidal flat	0.6	0.75	0.75	0.95	0.80	0.55	0.3
Urban land	0	0	0	0	0	0	0
Rural residential area	0	0	0	0	0	0	0
Other construction lands	0	0	0	0	0	0	0
Bare ground	0.1	0.3	0.4	0.6	0.5	0.6	0

**Table 3:** Area and dynamic degree of land use types from 2000 to 2020.

Type	Time	Cultivated Land	Forest Land	Grassland	Water Area	Construction Land	Unused Land
Land use area (km^2^)	2000	21,982.32	26,734.42	7401.01	765.44	475.76	8.81
	2005	21,702.40	26,965.22	7273.97	851.29	569.00	5.88
	2010	21,549.24	27,534.04	6232.97	1079.67	966.82	5.03
	2015	21,324.01	27,508.05	6223.03	1099.26	1208.40	5.01
	2020	21,172.15	27,091.70	5723.29	1130.90	2244.94	4.78
land use dynamics (%)	2000–2005	−0.25	0.17	−0.34	2.24	3.92	−6.66
	2005–2010	−0.14	0.42	−2.86	5.37	13.98	−2.89
	2010–2015	−0.21	−0.02	−0.03	0.36	5.00	−0.09
	2015–2020	−0.23	−0.01	−1.04	0.11	9.62	−0.78

**Table 4:** Transfer area of land use types from 2000 to 2020.

Time Interval	Land Use Type	Transfer Area (km^2^)					
		Cultivated Land	Forest Land	Grassland	Water Area	Construction Land	Unused Land
2000–2005	Cultivated land	21,327.63	409.68	125.09	28.56	91.30	0.06
	Forestland	229.25	26,403.34	41.65	49.71	10.46	0.03
	Grassland	138.44	148.43	7106.19	5.05	2.87	0.00
	Water area	3.55	1.80	0.80	758.31	0.97	0.01
	Construction land	2.71	1.84	0.23	7.59	463.38	0.01
	Unused land	0.91	0.02	0.02	2.09	0.02	5.76
2005–2010	Cultivated land	20,682.45	397.96	176.32	94.07	351.91	0.03
	Forestland	356.06	26,405.57	54.98	93.29	54.99	0.01
	Grassland	489.76	722.02	6000.26	42.15	19.74	0.00
	Water area	5.50	4.47	0.36	838.07	2.90	0.00
	Construction land	15.68	3.43	0.69	11.90	537.29	0.02
	Unused land	0.06	0.35	0.34	0.16	0.01	4.97
2010–2015	Cultivated land	21,091.72	177.87	56.20	12.06	211.77	0.05
	Forestland	167.21	27,297.49	27.04	11.51	30.38	0.01
	Grassland	55.67	27.34	6138.56	4.17	7.17	0.01
	Water area	4.48	3.04	0.81	1069.18	2.17	0.00
	Construction land	5.36	1.82	0.34	2.34	956.96	0.00
	Unused land	0.06	0.02	0.00	0.00	0.01	4.93
2015–2020	Cultivated land	19,245.73	1129.00	307.04	73.22	574.36	0.37
	Forestland	1215.54	26,025.26	161.61	31.25	68.88	0.16
	Grassland	519.97	255.08	5418.30	10.33	18.27	0.04
	Water area	38.55	58.18	8.37	979.39	15.05	0.02
	Construction land	55.28	25.00	4.35	10.82	1113.31	0.06
	Unused land	0.47	0.13	0.00	0.18	0.06	4.16
2000–2020	Cultivated land	18,974.79	1298.66	395.10	168.89	1150.71	0.26
	Forestland	1116.07	25,171.94	163.95	130.45	145.95	0.13
	Grassland	947.90	1005.74	5337.36	60.29	49.21	0.01
	Water area	21.63	10.00	2.48	721.50	9.94	0.00
	Construction land	13.61	6.29	0.62	21.45	433.93	0.07
	Unused land	0.98	0.49	0.34	2.63	0.05	4.32

**Table 5:** Area and ratio of each HQ level from2000 to 2020.

HQ Level	Value Interval	2000 Year		2005 Year		2010 Year		2015 Year		2020 Year	
		km^2^	%	km^2^	%	km^2^	%	km^2^	%	km^2^	%
Low	0–0.39	484.57	0.84	574.88	1.00	971.86	1.69	1213.42	2.12	1804.01	3.14
Medium-low	0.39–0.59	15,764.62	27.48	15,553.67	27.11	15,491.45	27.00	15,393.03	26.83	15,271.67	26.62
Medium	0.59–0.79	12,127.69	21.14	11,907.81	20.76	10,650.68	18.57	10,508.93	18.32	10,033.48	17.49
Medium-high	0.79–0.99	1820.44	3.17	1802.00	3.14	2005.20	3.50	2045.88	3.57	2286.50	3.99
High	0.99–1	27,170.44	47.36	27,529.40	47.99	28,248.57	49.24	28,206.51	49.17	27,972.12	48.76

**Table 6:** Statistics on the area of difference in habitat quality from 2000 to 2020.

Area	The Proportion of Each Type of Area (%)				
	Significant Degradation	Slight Degradation	Stable	Slight Improvement	Significant Improvement
TGRA	3.00	47.19	25.11	22.60	2.10
Head region of TGRA	0.26	7.49	9.27	2.42	0.22
Middle region of TGRA	1.35	27.15	14.10	18.56	1.57
Tail region of TGRA	1.39	12.55	1.74	1.62	0.31

## Data Availability

The datasets generated during and/or analyzed during the current study are available from the corresponding author on reasonable request.
